# Consent for Biobanking: The Legal Frameworks of Countries in the BioSHaRE-EU Project

**DOI:** 10.1089/bio.2015.0123

**Published:** 2016-06-01

**Authors:** Jane Kaye, Linda Briceño Moraia, Liam Curren, Jessica Bell, Colin Mitchell, Sirpa Soini, Nils Hoppe, Morten Øien, Emmanuelle Rial-Sebbag

**Affiliations:** ^1^ HeLEX Centre for Health, Law and Emerging Technologies, Nuffield Department of Population Health, University of Oxford, Oxford, United Kingdom.; ^2^ Academic Medical Centre of Helsinki (AMCH) Biobank, Helsinki, Finland.; ^3^ CELLS—Centre for Ethics and Law in the Life Sciences, Leibniz, Universitaet Hannover, Hannover, Germany.; ^4^ NTNU Norwegian University of Science and Technology, Trondheim, Norway.; ^5^ INSERM UMR 1027 Département d'épidémiologie et de santé publique, Faculté de Médecine, Université Paul Sabatier—Toulouse III, Toulouse, France.

## Abstract

Currently, there is no single, Europe-wide regulation of biomedical research using human samples and data. Instead, the law that applies spans a number of areas of law, such as data protection, clinical trials, and tissue regulation. In the absence of harmonized regulation, there is considerable scope for national legal variation. This article analyzes the legislative frameworks that apply to biobanking activities to identify differences in legal requirements between the BioSHaRE-EU project countries: Finland, France, Germany, the Netherlands, Norway, and the United Kingdom. This article highlights the primary role of consent and accompanying governance mechanisms, such as research ethics committee oversight, which enable consent exemptions in the context of research. Our analysis identifies a complicated legal landscape, whereby broadly similar provisions are contained in varied sources of law in each jurisdiction. The challenge for researchers is locating the applicable legal provisions within each national legal framework.

## Introduction

Biomedical research in Europe relies on the collection of human tissue and the subsequent extraction, manipulation, and linkage of associated data. The regulation of this activity is largely carried out at a national level as currently there is no single, Europe-wide piece of regulation for biomedical research that applies to both human samples and data. Research Ethics Committees (RECs) play a crucial role in determining whether research should go ahead within the different national legal frameworks. Some countries have chosen to sign and ratify the international Council of Europe Convention on Human Rights and Biomedicine^1^ and its protocols relating to research uses of human tissue, but member states, such as the United Kingdom, have not.

Instead, the law that applies to biomedical research at the European level spans a number of areas of law, such as data protection (Data Protection Directive 95/48 EC and the new General Data Protection Regulation) clinical trials (Regulation EU No. 536/2014) and tissue regulation (Tissues and Cells Directive 2004/23/EC). This year, two directives that apply to tissue within Europe came into force,^2,3^ but neither apply to tissue that is used for research purposes. The regulatory landscape is undergoing considerable reform with the introduction of the Clinical Trials Regulation (Regulation EU No. 536/2014) and the General European Data Protection Regulation (GDPR), perhaps signaling a move toward a greater degree of harmonization between member states (although with a “margin of appreciation” for member states as to how these are implemented).

The EU Parliament, Commission, and Council have now agreed to a General Data Protection Regulation that will harmonize aspects of data protection across Europe. This regulation will become effective in 2018, and in the interim, the Data Protection Directive and existing national laws still apply. As a regulation, the GDPR will be directly binding and carries over many of the same principles as the Data Protection Directive. For research and biobanking, broad consent is generally acceptable, where it is not possible to be specific about the purposes of the research. Member states will also be able to legislate for processing of health data for research purposes without consent, so long as safeguards are in place, and the regulation requires safeguards, such as pseudonymization, to be used, where possible, in research.

In the absence of uniform European regulation of biobanks, research participants or donors to biorepositories may consent using different documents in different jurisdictions for different types of research. This has implications for further research because it prevents the creation of a single harmonized data set that can be used easily across borders. Equally, not all participants across Europe will receive the same level and type of information on potential uses of their samples and data and any risks involved. BioSHaRE-EU is one of a number of collaborative projects aiming to harmonize and standardize biobanking across Europe. Other European and international examples include P3G Public Population Project in Genomics and Society, BBMRI-ERIC EU (Biobanking and BioMolecular resources Research Infrastructure), and International Cancer Genome Consortium (ICGC). This article will comparatively analyze the legislative frameworks for consent and biobanking activity in the six original BioSHaRE-EU countries, which each having well-established biobanks: Finland, France, Germany, the Netherlands, Norway, and the United Kingdom.

## Methods

It would be logical to presume that having national legal frameworks governing this activity would lead to considerable differences in the legal requirements across Europe. To test this assumption, we analyzed the legislative frameworks that applied to biobanking activities in Finland, France, Germany, the Netherlands, Norway, and the United Kingdom to identify the differences between them. Biobanks based in these countries are part of the BioSHaRE-EU project, which aims to develop tools to enable large-scale analysis, involving a number of biobanks located in different EU member states.^4^ These countries were the original members of the BioSHaRE-EU project and now have well-established biobanks. As such, we have chosen to focus our investigation on these five countries, and the legislation in each of these jurisdictions applies to consent for (1) research, (2) biobanking, (3) the use of samples, and (4) data for research purposes.

To conduct our comparative legal analysis, representative experts from each jurisdiction conducted analysis of their jurisdictional legislative frameworks and applied information about the relevant country-specific provisions to the issue of consent. Where there is jurisdictional regulation relating to specific consent, we include details of such provisions; for the remaining countries, there is an absence of specific consent requirements, and the alternative approach will be described.

## Results

Our study showed that there was remarkable uniformity in the consent processes for samples and data, but in each of these countries, this was achieved using different legal means, which will be detailed below.

## Discussion

### Finland

Finland enacted the Medical Research Act in 2000 to implement the EU Clinical Trials Directive and set norms for all medical research.^5^ The Medical Research Act sets rigorous criteria for informed consent and requires, among other things, ethics committee approval before research taking place (s. 3). The act was seen, inter alia, to hinder the feasibility of broad consent for medical research. To improve the research framework, Finland enacted specific legislation for biobanking^6^ in 2013, which provides the legal framework for the collection, storage, and processing of samples and sets out the rights of individuals and arrangements necessary to protect personal information.^7^

Under this Biobank Act, a biobank's entitlement to process samples is primarily based on a broad and informed consent: The participant receives extensive information on the nature and objectives of the biobank and can track the use of samples and data (s. 11). A special feature of the act is to allow the transfer of old clinical samples and research samples into a biobank following a special procedure notifying individuals and providing an opt-out to inclusion of material in the biobank. Samples and data in a Finnish biobank shall be shared within the research community, and the results shall enrich the biobank.

The Act on the Medical Use of Human Organs and Tissues^8^ provides additional exemptions for the secondary use of clinically obtained samples for medical research and to be used in biobanks without consent (s. 20). Secondary use may take place, subject to an ethical review, if the person is deceased. Sometimes a permit from the National Supervisory Authority for Welfare and Health is required. This will be granted only if an REC has also issued a favorable opinion on the matter (s. 21a).

For the processing of personal data, the Personal Data Act^9^ is a *lex generalis* and will apply unless specific provisions are found elsewhere in the law. Thus, the Personal Data Act complements other acts. This act directly implemented the provisions of the Data Protection Directive 95/46/EC. Under the act, consent is generally required under section 8, but processing is lawful for scientific research if “the research cannot be carried out without identifying the person and the consent of the data subjects cannot be obtained owing to the quantity of the data, their age or another comparable reason” [para. 14 (1)]. These provisions for data mirror those of the directive and therefore are in conformity with requirements across Europe. The Biobank Act is regarded as *lex specialis*, and thus, many of its provisions prevail over the more general Personal Data Act.

### France

Under the French legal framework for the storage and use of human biological samples, several pieces of legislation must be respected: the bioethics law,^10^ the law on biomedical research,^11^ and the law regarding data protection.^12^ The legislation does not refer to biobanks *per se* (the term “biobank” does not exist in any legal text) but instead to the collection of biological samples.^13^ In addition to this definition, the Public Health Code describes two kinds of activities that could be developed by researchers collecting samples: collection of samples by researchers for the needs of their own research programs and collection of samples for transfer (i.e., biobanking).

Researchers must obtain informed consent from the donor of the biological material before starting any research activity. Written consent must be obtained based on clear information, and the consent can always be withdrawn (Article L 1211-2 of the Public Health Code). Multiple consents have to be given for the process of biobanking: for the storage, handling, use, and research purpose for which the participant has given its tissues and cells and associated data. Although samples and data are covered by two pieces of legislation, the consent has to mention the processing of associated personal data for research purposes.

Alternatively, for previously collected samples, a specific procedure has been set up in the bioethics law to facilitate their use (Article LL1211-2, Public Health Code) through the implementation of an opt-out procedure. This opt-out procedure does not apply when genetic analyses are performed. In this case, a written informed consent is required. The institutions in charge of the legal and ethical assessments of sample collections are, on the one hand, the Committee of Persons' Protection (CPP, Ethics committee) and, on the other hand, the Ministry of Research. Both must be consulted, and they assess the conformity of the research protocol with research/bioethics laws.

Health data, including genetic data, are considered as sensitive data according to the definition of the Data Protection Act (art. 8-1) and in accordance with the 95/46/EC Directive. Regarding the processing of genetic information, in order for it to be lawful, it is necessary to obtain the express consent of the individuals whose data are to be collected, as stated in Art 56: “where the research requires the collection of identifying biological samples, the informed and express consent of data subjects must be obtained prior to the implementation of data processing.” In principle, collection, processing, and transfer abroad of such data are forbidden, except if the data subject has given his or her express consent or for necessary medical research. The transfer of personal data abroad is restricted to foreign countries, ensuring a sufficient level of protection. The French National Data Protection Supervisory Authority is called “Commission Nationale Informatique et Liberté” (CNIL); it is in charge of the supervision of the personal data collection and uses.

### Germany

There is no specific legislation concerning biomedical research as a whole within Germany, but instead, there are three different pieces of legislation that in combination cover this area. These are (1) the Act on Medical Devices (Medizinproduktegesetz [MPG]) adopted in 1994, (2) the fifth Amendment of the Act on Pharmaceutical Products (Arzneimittelgesetz) adopted in 1995, and (3) the Code of Deontology (“Ärztliche Berufsordnung”), which, in the German states, is a legally binding instrument. All these instruments have requirements that research should be reviewed by an REC, which is also a requirement of the European Clinical Trials Regulations.

In addition, there are more general constitutional rights under the German Basic Law (Grundgesetz) pertaining to matters, such as individual personality, dignity, and integrity.^14^ This provision requires the prior consent of an individual for the donation of tissue for research purposes,^15–17^ which should be informed by the “purpose, significance, and implications of the encroachment,” that is, whether the samples are taken for the specific purpose of research and biobanking.^15^ However, consent is not necessary for downstream use of lawfully procured tissue as between biobanks and researchers. This is because tissue, when separated from the body, is treated as a form of property under German law.^15,17,18^ In this framework, the transfer of the tissue between parties is treated by way of contractual arrangements, such as assignments and licenses, as any other form of property.^19^ The Gesetz über Qualität und Sicherheit von menschlichen Geweben und Zellen (Act on Quality and Safety of Human Tissues and Cells, also known as Human Tissue Act, BGBl. I 2007, 1574) implemented the EU Directive 2004/23/EC^20^ in German law (17, p. 92).

The law in Germany differs from other European countries as it often has higher protections than those stipulated in European law. An example of this is the consent requirements for the use of data in research. Consent needs to be given in writing unless special circumstances warrant another form. The EU Data Protection Directive is implemented in a federal act (BDSG) and fragmented across individual state and canonical legislation in Germany.^21^ These instruments distinguish between public and private entities and do not apply to anonymized data. The BDSG definition of sensitive data expressly includes health data [s. 3(9)]. All data use ought to take place under the umbrella of valid informed consent. Derogations from this principle are possible in cases where the scientific value of the exercise outweighs the individuals' right to opt out or where research cannot otherwise not be undertaken at all or only at disproportionate effort [ss. 13(2) and 28(6)]. This is a unique feature of the German law as it is a higher standard than the requirements of 95/46/EC Directive. It is possible that this “special circumstance” may exist in the field of scientific research [s. 28(6)4], but generally, consent shall be recorded in writing, where special categories of personal data, such as health data, are collected, processed, or used [s. 3(9)].

While there are no biobank-specific laws that operate in Germany, an opinion on human biobanks was published by the German Ethics Council in June 2010.^15^ There has been a public discussion for a number of years on the benefits of implementing specific biobank legislation, but to date, this has not happened. In 2015, a biobank law for Germany was drafted to address the deficit in the law, but currently, this is just a proposal.^22^

### Netherlands

In the Netherlands, there is no general regulation for biomedical research or specific regulation for biobanking,^23^ although the Dutch Council of the Federation of Medical Scientific Societies (Federatie Van Medisch Wetenschappelijke Verenigingen) has set out a Code of Conduct for medical research. Instead, there is a detailed legal framework, spanning a number of sources, for use of tissue (including sperm, ova, and fetal tissue) and data. As with other jurisdictions, there is the requirement that research must be approved by an REC.

In terms of samples, the general principle within the Dutch Civil Code, (Burgerlijk Wetboek), via provisions contained in the Medical Treatment Contracts Act (*Wet op de geneeskundige behandelingsovereenkomst*), is that consent is required. However, these regulations permit the use of human tissue for “medicostatistical or other medicoscientific research.” There are two conditions for such research taking place: the “patient,” from whom the tissue was extracted, must not have objected to the research and any resulting data from the use of this tissue must not be capable of being “traced back to the person from whom they originated” (Art. 7:467). The Human Tissue Requirements Decree of 2006 (*Eisenbesluit Lichaamsmateriaal*) also sets out requirements with respect to the safety and quality of human tissues used in medical treatment. This legislation also contains provisions regarding data derived from samples, including institutional responsibilities to ensure that data accessible by third parties, such as genetic information, can no longer be traced to individuals (Art. 9.1).

Like all other member states in Europe, the Netherlands has implemented the provisions of the Data Protection Directive 95/46/EC. The Personal Data Protection Act of 2000 (Wet bescherming persoonsgegevens) implements the EU Directive into Dutch law. Personal data can be used for the purpose of scientific research, without the data subject's consent, so long as (1) the research “serves a public interest,” (2) is necessary for such research, (3) would be “impossible or would involve a disproportionate effort to ask for express consent,” and (4) that “sufficient guarantees” are provided to ensure there are no disproportionately adverse effects on individuals' privacy [Art. 23(2)].

### Norway

In common with Finland, Norway also has specific legislation for the regulation of medical research. In Norway, the research use of both personal health data and samples is regulated by the same legislative instrument: The Health Research Act (Helseforskningsloven—hereafter the “HRA”).^24^ This single comprehensive piece of legislation contains specific provisions relating to research biobanks and research involving human biological material and requires the storage and processing of material in research biobanks to be carried out with respect for the donor's wishes. Patients must be informed in advance that their biological material may be used for research and they have the option to refuse to be involved in research on human biological material. Moreover, an electronic register has been established with the patients who have stated that they do not wish their biological material to be used for research (s. 28). Under the act, unless specific legal authority or another valid legal basis exists, the collection, storage, and processing of human biological material and data for research purposes require consent by the donor, which should be (1) voluntary, (2) express, and (3) informed (s. 12). If previously collected material and data are subject to a different, wider, or new use, which does not fall within the scope of the original consent, then new voluntary, express, and informed consent must be obtained.

However, it is not always strictly necessary to gain the explicit consent of the data subject to process personal health data for research purposes if the specific conditions set out in the Health Research Act are met (s. 35). The REC may decide that such data can or shall be handed over by health personnel for use in research if, and only if, the research in question is of significant interest to society and the data subject's welfare and integrity are ensured. The REC may also specify further conditions for the use of the data and the rules on confidentiality pursuant to section 7 of the act will apply to the recipient party accordingly.

Moreover, where it is impossible or very difficult to obtain new consent, the Regional Committee for Medical and Health Research Ethics (REC) may grant an exemption from this requirement (s. 15). The REC may specify conditions for the new or changed use. Uses of anonymized material do not require consent (s. 20). Any person who has given his or her consent pursuant to sections 11–13 may withdraw such consent at any time. In this case, the person who gave such consent has the right to require that the biological material, as well as any health and personal data collected together, is destroyed, erased, or returned unless the material has been anonymized or is already being used in scientific work.

### United Kingdom

Like the Netherlands and Germany, the United Kingdom adopts a similarly fragmented legislative approach to the regulation of tissues and data in research. Unlike Finland and Norway, there is no single piece of regulation for biomedical research or biobanking. The distinction between tissue and data that are found in other jurisdictions is also a feature of U.K. legislation.

In relation to tissue, the Human Tissue Act 2004 regulates the removal, storage, use, and disposal of human tissue for scheduled purposes via the specifically created regulatory body, the Human Tissue Authority (HTA). One of the HTA statutory functions under the act is to issue Codes of Practice, which should give guidance to researchers interested in carrying out activities regulated by the HTA. Even if noncompliance with these Codes of Practice is not in itself an offense under the HT Act, it could influence licensing decisions made by the HTA.

Whereas the data protection legislation provides for processing of data for research purposes without consent in certain circumstances, the Human Tissue Act holds the “appropriate consent” of individuals as a core feature. Obtaining valid consent presupposes that there is a process in which individuals (including their families where appropriate) may discuss the issue fully, ask questions, and make an informed choice. That said, tissue from living donor that has been properly anonymized and has received appropriate REC approval can be used in research without the consent of the donor.^25^ A unique feature of the U.K. legal framework regarding access to samples is that an HTA license is required whenever relevant material is held for research purposes (s. 16 HTA). However, there are some exceptions, where the license is not required.

The Data Protection Act of 1998 implements the EU Data Protection Directive and regulates the “processing of personal data,” also allowing processing for research purposes (s. 33). In the United Kingdom, it is not necessary to gain the explicit consent of the data subject to process sensitive data for research purposes if conditions set out in a Processing of Sensitive Personal Data Order^26^ are met. This requires the processing to be in the substantial public interest, necessary for research purposes, that it does not support measures or decisions with respect to any particular data subject otherwise than with the explicit consent of that data subject and that it does not cause, nor is likely to cause, substantial damage or substantial distress to the data subject or any other person.^27^

## Conclusions

Each of the BioSHaRE-EU project countries has provisions that apply to the use of samples and data for research purposes, but these are contained in different sources of law in each jurisdiction. This is because there is no single European legislative instrument equivalent to the European Data Protection Directive 45/46/EC that regulates the use of human tissue in research or biomedical research in general. The result is that across the member states, a range of national laws apply to the use of human tissue in research, including different licensing regimes, individual rights, biobank-specific laws, and research-specific laws.

The key difference is the legal basis for consent provisions, which may be found in constitutional law (Germany), Civil Code (the Netherlands), statute (Finland and Norway), or a combination of legislation and regulations (the United Kingdom and France). Some countries rely on general law to cover medical research and biobanking combined with more specific codes of practice or opinions from national bodies (Germany, the Netherlands, and the United Kingdom). A common trend is to maintain different legal regimes for samples and data. However, Finland and Norway have each implemented law that unifies consent requirements for data and samples. French legislation cross-references between biomedical research instruments and data protection rules.

Consent is the primary justification and requirement for research or biobank use of samples across these BioSHaRE-EU states. For example, the Finnish Biobank Act places consent at the heart of biobank regulation and the primary basis for use of samples by biobanks (s. 11). This law explicitly grants individuals the right to change their consent to types of research at any time (s. 12) and contains provisions for biobanks to maintain a “consent register” to record any restrictions on use of individual samples [s. 22(3)]. However, almost all nations provide exemptions to the requirement for consent for research use of samples, provided certain conditions are met. The laws of the Netherlands, France, Norway, the United Kingdom, and Finland provide legal pathways for the transfer of clinical samples to biobanks for medical research without consent. In all the BioSHaRE-EU countries, REC approval is required for all research that is carried out on human beings as within Europe they are “the key decision-makers in reviewing and allowing a research protocol.”^28^ However, there are different REC approval procedures across the BioSHaRE-EU jurisdictions,^29^ and the only common feature is that the REC or, in France, the Ministry of Research must approve the research plan before any research is undertaken. In the literature, it has been highlighted that “broad consent” is effectively “consent for governance” by others as judgments about appropriate uses of data and samples “often fall to researchers, advisory boards, or RECs, who must make decisions on behalf of research participants.”^30^ The discretionary nature of REC judgments has the potential to amplify existing local differences within this fragmented legal system.

Of the BioSHaRE-EU jurisdictions analyzed in this article, only Germany lacks an exemption to the requirement for consent to research using samples, although if consent has been provided to initial research use, then secondary use of samples in Germany is instead governed by property law, contracts, and licenses. German law's insistence on consent for research use of samples also contrasts with the position for research using personal data within the terms of the EU Data Protection Directive since all five BioShaRE-EU jurisdictions allow the use of identifiable personal data for research purposes without consent in certain circumstances.

Our analysis has illustrated the importance of consent in biobanking and medical research across the BioShaRE-EU jurisdictions as consent requirements and regulatory oversight of secondary research without consent are stipulated in the law of every BioShaRE-EU country. The key difference across the countries is where these requirements are found in the national legal framework. Some nations set them out in biobank-specific legislation; for the others, they are contained in more general areas of law. As a result, there are some minor variations in consent requirements, in particular Finland and Norway have more specific biobank legislation and more sophisticated consent models. The implications of the GDPR and the provision for derogation between member states may mean that this fragmented approach is likely to continue. Therefore, the challenges for researchers are to identify the appropriate jurisdiction and locate the applicable legal provisions on consent within each country. Platforms, such as P3G^31^ and hSERN,^32^ are important in helping researchers access and navigate this complex legal landscape.

## References

[1] Convention for the protection of human rights and dignity of the human being with regard to the application of biology and medicine: Convention on human tights and biomedicine, Oviedo, 4.IV.1997 http://conventions.coe.int/treaty/en/Treaties/Html/164.htm Accessed 427, 2016

[2] Directive 2015/565 as regards certain technical requirements for the coding of human issues and cells. http://eur-lex.europa.eu/legal-content/EN/TXT/?uri=CELEX:32015L0565 Accessed 427, 2016

[3] Directive 2015/566 as regards the procedures for verifying the equivalent standards of quality and safety of imported tissues and cells. http://eur-lex.europa.eu/legal-content/EN/TXT/?uri=CELEX:32015L0566 Accessed 427, 2016

[4] BioSHaRE is a consortium of leading biobanks and international researchers from all domains of biobanking science. www.bioshare.eu Accessed 427, 2016

[5] Medical Research Act (488/1999, 295/2004, 794/2010). www.finlex.fi/en/laki/kaannokset/1999/en19990488.pdf

[6] Biobank Act (688/2012).www.finlex.fi/en/laki/kaannokset/2012/en20120688.pdf Accessed 427, 2016

[7] Soini S Finland on a road towards modern biobanking legislation. Eur J Health Law 2013;20:3:289–294 23984494 10.1163/15718093-12341278

[8] Act on the Medical Use of Human Organs and Tissues No. 101/2001 (as amended). www.finlex.fi/en/laki/kaannokset/2001/en20010101.pdf Accessed 427, 2016

[9] Personal Data Act (523/1999). www.finlex.fi/en/laki/kaannokset/1999/en19990523.pdf Accessed 427, 2016

[10] Bioethics Law no. 2011-814, July 7th, 2011 modifying the previous Law of 2004 published in article 12 of the French Official journal on August 7th, 2004 included in the French Public Health Code.www.legifrance.gouv.fr/eli/loi/2011/7/7/2011-814/jo/texte Accessed 427, 2016

[11] Law no. 2004-806, August 9th, 2004 related to the Public Health Politics included in the French Public Health Code. www.legifrance.gouv.fr/affichTexte.do?cidTexte=JORFTEXT000000787078 Accessed 427, 2016

[12] Data Protection Act no. 78-17, “Loi Informatique et Liberté” of 6 January 1978, consolidated version of 2009. www.legifrance.gouv.fr/affichTexte.do?cidTexte=JORFTEXT000000886460 Accessed 427, 2016

[13] Public Health Code, Art L. 1243-3.www.legifrance.gouv.fr/affichCode.do;jsessionid=079B5532E0C0B5428C0E693AE3EC5B45.tpdila10v_2?cidTexte=LEGITEXT000006072665&dateTexte=22220222 Accessed 427, 2016

[14] Basic Law for the Federal Republic of Germany in the revised version published in the Federal Law Gazette Part III, classification number 100-1, as last amended by the Act of 11 July 2012 (Federal Law Gazette I p. 1478) Arts. 1 and 2 (read in conjunction with Federal Constitutional Court (*BVerfG*) case law interpreting these articles).www.gesetze-im-internet.de/englisch_gg/englisch_gg.html Accessed 427, 2016

[15] Deutscher Ethikrat. 2010 Human biobanks for research: Opinion. www.ethikrat.org/files/der_opinion_human-biobanks.pdf Accessed 427, 2016

[16] Lenk C, Hoppe N, Beier K, Wiesemann C Human Tissue Research. Oxford, United Kingdom: Oxford University Press; 2011

[17] Hoppe N Bioequity—Property and the Human Body. Farnham: Ashgate; 2009

[18] Björkman B, Hansson S Bodily rights and property rights. J Med Ethics 2006;32:209–214 16574874 10.1136/jme.2004.011270 PMC2565785

[19] Civil Code in the version promulgated on January 2, 2002 (Federal Law Gazette [Bundesgesetzblatt] I page 42, 2909; 2003 I page 738), last amended by Article 4 para. 5 of the Act of October 1, 2013 (Federal Law Gazette I page 3719) para. 854–1296. www.gesetze-im-internet.de/englisch_bgb/englisch_bgb.html Accessed 427, 2016

[20] EU Directive 2004/23/EC of the EU Parliament and of the Council of 31 March 2004, on setting standards of quality and safety for the donation, procurement, testing, processing, preservation, storage and distribution of human tissues and cells. OJEU L 102/48

[21] Federal Data Protection Act in the version promulgated on January 14, 2003 (Federal Law Gazette I p. 66), as most recently amended by Article 1 of the Act of 14 August 2009 (Federal Law Gazette I p. 2814). www.gesetze-im-internet.de/englisch_bdsg/englisch_bdsg.html Accessed 427, 2016

[22] Gassner U, Kersten J, Lindemann M, Lindner FJ, Rosenau H, Schmidt am Busch B, Schroth U, Wollenschläger F Biobank Act: The Augsburg-Munich Draft (AME-BiobankG). Tübingen: Mohr Siebeck; 2015

[23] Bovenberg JA Rights and entitlements in human tissue and cells, 77. In: Beier K, Schnorrer S, Hoppe N (eds.). The Ethical and Legal Regulation of Human Tissue and Biobank Research in Europe. Proceedings of the Tiss.EU Project. Göttingen: Universitätsverlag Göttingen; 2011

[24] The Health Research Act 2008 (Helseforskningsloven). http://app.uio.no/ub/ujur/oversatte-lover/data/lov-20080620-044-eng.pdf Accessed 427, 2016

[25] Human Tissue Authority. Code of Practice 9 Research. 2014 www.hta.gov.uk/guidance-professionals/codes-practice/code-practice-9-research

[26] The Data Protection (Processing of Sensitive Personal Data) Order 2000, Statutory Instrument 2000 No. 41. www.legislation.gov.uk/uksi/2000/417/contents/made Accessed 427, 2016

[27] Kaye J, Briceno Moraia L, Mitchell C, Bell J, Bovenberg J, Tasse AM, Knoppers BM Access governance for Biobanks: The case of the BioSHaRE-EU cohorts. Biopreserv Biobank 2016;14:201–206 27183185 10.1089/bio.2015.0124 PMC5939924

[28] Gottweis H, Kaye J, Bignami F, et al. Biobanks for Europe; A Challenge for Governance. Luxembourg: European Commission; 2012 DOI:10.2777/68942

[29] Veerus P, Lexchin J, Hemminki E Legislative regulation and ethical governance of medical research in different European Union countries. J Med Ethics 2014;40:409–413 23665856 10.1136/medethics-2012-101282

[30] Kaye J The tension between data sharing and the protection of privacy in genomics research. Ann Rev Genomics Hum Genet 2012;13:415–431 22404490 10.1146/annurev-genom-082410-101454 PMC4337968

[31] Public Population Project in Genomics and Society. www.p3g.org Last accessed 427, 2016

[32] Human Sample Exchange Regulation Navigator. www.hsern.eu Accessed 427, 2016

